# Prevalence of healthy aging among community dwelling adults age 70 and older from five European countries

**DOI:** 10.1186/s12877-022-02755-8

**Published:** 2022-03-02

**Authors:** Simeon Schietzel, Patricia O. Chocano-Bedoya, Angelique Sadlon, Michael Gagesch, Walter C. Willett, Endel J. Orav, Reto W. Kressig, Bruno Vellas, René Rizzoli, José A. P. da Silva, Michael Blauth, John A. Kanis, Andreas Egli, Heike A. Bischoff-Ferrari

**Affiliations:** 1 Department of Aging Medicine and Aging Research, University of Zurich and University Hospital Zurich, Switzerland, Zurich, Switzerland; 2 Center on Aging and Mobility (CAM), University of Zurich, University Hospital Zurich, and City Hospital Zurich, Waid, Switzerland; 3 Institute of Primary Health Care (BIHAM), University of Bern, Bern, Switzerland; 4 Population Health Laboratory (#PopHealthLab), University of Fribourg, Fribourg, Switzerland; 5 Department of Biostatistics, Harvard T. H. Chan School of Public Health, Boston, Massachusetts USA; 6 University Department of Geriatric Medicine Felix Platter and University of Basel, Basel, Switzerland; 7 Gérontopôle de Toulouse, Institut du Vieillissement, Center Hospitalo-Universitaire de Toulouse, Toulouse, France; 8 UMR INSERM 1027, University of Toulouse III, Toulouse, France; 9 Division of Bone Diseases, Geneva University Hospitals and Faculty of Medicine, Geneva, Switzerland; 10 Centro Hospitalar e Universitário de Coimbra, Coimbra, Portugal; 11 Coimbra Institute for Clinical and Biomedical Research (iCBR), Faculty of Medicine, University of Coimbra, Coimbra, Portugal; 12 Department for Trauma Surgery, Medical University of Innsbruck, Innsbruck, Austria; 13 Centre for Metabolic Bone Diseases, University of Sheffield Medical School, Sheffield, UK; 14 University Clinic for Aging Medicine, City Hospital Zurich, Zurich, Waid Switzerland

**Keywords:** Healthy Aging, DO-HEALTH, Older, Senior

## Abstract

**Background:**

To compare the prevalence of healthy aging among adults age 70 and older from 5 European countries recruited for the DO-HEALTH clinical trial. Participants were selected for absence of prior major health events.

**Methods:**

Cross-sectional analysis of DO-HEALTH baseline data. All 2,157 participants (mean age 74.9, SD 4.4; 61.7% women) were included and 2,123 had data for all domains of the healthy aging status (HA) definition. HA was assessed based on the Nurses` Health Study (NHS) definition requiring four domains: no major chronic diseases, no disabilities, no cognitive impairment (Montreal Cognitive Assessment, MoCA ≥25), no mental health limitation (GDS-5 <2, and no diagnosis of depression). Association between HA and age, BMI, gender, and physical function (sit-to-stand, gait speed, grip strength) was assessed by multivariate logistic regression analyses adjusting for center.

**Results:**

Overall, 41.8% of DO-HEALTH participants were healthy agers with significant variability by country: Austria (Innsbruck) 58.3%, Switzerland (Zurich, Basel, Geneva) 51.2%, Germany (Berlin) 37.6%, France (Toulouse) 36.7% and Portugal (Coimbra) 8.8% (p <0.0001). Differences in prevalence by country persisted after adjustment for age. In the multivariate model, younger age (OR = 0.95, 95% CI 0.93 to 0.98), female gender (OR = 1.36, 95% CI 1.03 to 1.81), lower BMI (OR = 0.94, 95% CI 0.91 to 0.96), faster gait speed (OR = 4.70, 95% CI 2.68 to 8.25) and faster performance in sit-to-stand test (OR = 0.90, 95% CI 0.87 to 0.93) were independently and significantly associated with HA.

**Conclusions:**

Despite the same inclusion and exclusion criteria preselecting relatively healthy adults age 70 years and older, HA prevalence in DO-HEALTH varied significantly between countries and was highest in participants from Austria and Switzerland, lowest in participants from Portugal. Independent of country, younger age, female gender, lower BMI and better physical function were associated with HA.

**Trial registration:**

DO-HEALTH was registered under the protocol NCT01745263 at the International Trials Registry (clinicaltrials.gov), and under the protocol number 2012–001249-41 at the Registration at the European Community Clinical Trial System (EudraCT).

**Supplementary Information:**

The online version contains supplementary material available at 10.1186/s12877-022-02755-8.

## Background

For the five DO-HEALTH countries, the UN Population Division estimated in 2019 that the proportion of older adults will grow considerably. Between 2015 and 2050 the population age 65 and older was predicted to grow from 18% to 28.7% in Switzerland, from 18.8% to 29.4% in Austria, from 21.2% to 30% in Germany, from 18.9% to 27.8% in France and from 20.8% to 34.8% in Portugal [1]. The number of older adults with age-related chronic diseases will increase accordingly [2, 3]. Therefore, enabling more older adults to stay healthy and active longer is of major public health importance, and directly linked to reducing the burden of frailty and age-related chronic diseases [4].

However, effective older adult health promotion requires a clear definition of healthy aging, identification of risk factors and comparative analyses of different populations. Defining a healthy aging status (HA) is challenging due to the inherent multidimensionality of human health [5]. To date, there is no consensus on a definition. Numerous definitions exist, aiming to capture one or multiple dimensions of human health in older age. Approaches range from simple concepts like absence of diseases and disabilities [6] up to extensive evaluations of physical and mental diseases as well as cognitive and social functions [7–10]. In addition, definitions integrate self-rated health [11], quality of life [12], personal resources [13], well-being [14], socio-economic factors [15], personality traits [16], environment [17], resilience [18] and ability to adapt [19]. Furthermore, applied assessments differ vastly between studies. From 15 European studies assessing the HA dimension of disabilities [6, 9, 10, 17, 20–30], only two studies [9, 28] operationalized this HA domain in the same way. From 16 out of 25 European studies assessing the HA dimension of cognitive function [6, 9, 12–15, 18, 20, 22, 25–27, 29–32], only five studies applied the same cognitive assessment [10, 22, 26, 27, 29] and none of these studies used the same cutoff value.

Based on the requirements of a given HA definition, the prevalence of HA varies from 0.4% to 41% even in the same population, [33] and reaches up to 92% in one-dimensional definitions [34, 35]. With regard to important European studies on HA [6, 9, 10, 12, 15, 17, 24–27, 29] that focused on adults not younger than 60 and not older than 85 years of age, [9, 15, 27, 29] reported prevalence ranges from 1.6% to 49.3%. However, given the tremendous conceptual differences in HA definitions these results may not reflect differences in population state of health.

A well-validated concept is the Nurses’ Health Study (NHS) HA definition [6, 25, 30, 36–39], which has been linked to healthy survival after the age of 70 [36–38].

DO-HEALTH baseline data provided the opportunity to operationalize the NHS definition enabling application of the same multi-dimensional HA definition to older adults from five different countries. DO-HEALTH is a multicentre randomized trial to explore differences in HA in Europe among pre-selected relatively healthy adults age 70 years and older, living in the community, overall and by subgroups of age and by country. Further, we investigated cross-sectional correlates of HA in this sample of relatively healthy older adults.

## Methods

### Participants and study design

This is a cross-sectional analysis of baseline data collected in DO-HEALTH, a multi-center randomized controlled trial designed to test the effects of vitamin D, omega-3 and a home-exercise program among community-dwelling adults age 70 years and older (ClinicalTrials.gov Identifier: NCT01745263). The total population included 2157 community-dwelling older adults from Switzerland (Zurich, Basel, Geneva), Austria (Innsbruck), Germany (Berlin), France (Toulouse) and Portugal (Coimbra). By targeting 40% of participants who experienced a fall in the year prior to enrollment, the study aimed to also include pre-frail older adults. For inclusion in DO-HEALTH, participants had to be without major health events (i.e. cancer, angina pectoris, myocardial infarction, stroke, severe kidney or liver disease) in the five years prior to enrollment, a Mini Mental Status Examination (MMSE) score of at least 24 (the MMSE as the cognitive measure for inclusion in DO-HEALTH correlated with outcome measure MOCA in DO-HEALTH at the baseline exam by 0.495), and sufficient mobility to be able to come to the study center. The study was approved by the ethical and regulatory agencies of all 5 countries. Written informed consent was obtained from all participants prior to screening procedures.

### Definition of healthy aging status and operationalization in DO-HEALTH

We defined HA according to the definition used in the NHS,(36-38) which includes four domains: no major chronic diseases, no disabilities, no impairment in cognitive function and no mental health limitations. For an in-depth description of items and how they were operationalized in DO-HEALTH see Table 1 and Additional file 1.

1. no major chronic diseases

**Table 1:** Operationalization of the NHS definition of the healthy aging status phenotype in DO-HEALTH

NHS	DO-HEALTH
**No major chronic diseases**	
NHS questionnaire	Sangha
1. No type 2 diabetes	1. No type 2 diabetes
2. No congestive heart failure	2. No heart disease
3. No kidney failure	3. No kidney disease
4. No chronic obstructive pulmonary disease	4. No lung disease
5. No cancer other than non-melanoma skin cancer	5. No cancer other than non-melanoma skin cancer *
6. No myocardial infarction	6. No myocardial infarction *
7. No coronary artery bypass surgery, or angioplasty	7. No coronary intervention *
8. No stroke	8. No stroke *
9. No Parkinson’s disease	9. No Parkinson`s disease †
10. No multiple sclerosis	10. No multiple sclerosis †
11. No amyotrophic lateral sclerosis	11. No amyotrophic lateral sclerosis †
**No disabilities**	
SF-36	PROMIS
No limitation in:	No limitation in:
1. Bathing	1. Wash and dry your body
2. Dressing	2. Dress yourself, including shoelaces and buttons
3. Climbing one flight of stairs	3. Climb up five steps
4. Walking >1 mile or walking several blocks	4. Walk a block on flat ground
5. Moving a table	5. Stand up from an armless straight chair ±
6. Bowling or Golf	6. Get in and out of bed ±
7. Pushing a vacuum cleaner	7. Get on and off the toilet ±
No more than moderate limitations in:	No more than moderate limitations in:
8. Lifting or carrying groceries	8. Run errands and shop
9. Bending, kneeling, or stooping	9. Do chores such as vacuuming or yard work
10. Lifting heavy objects	10. Reach and get down a 2-Kg object from above head
11. Climbing several flights of stairs	11. Bend down and pick up items from the floor ±
**No impairment in cognitive function**	
TICS score >31	MoCA score >=25 §
**No mental health limitation**	
SF-36, MHI score >84/100	GDS-5 score < 2/5
How much of the time during the last month have you:	1. Do you feel pretty worthless the way you are now?
1. Felt so down in the dumps that nothing could cheer you up?	2. Are you basically satisfied with your life?
2. Been a happy person?	3. Do you often feel helpless?
3. Felt downhearted and blue?	4. Do you prefer to stay at home rather than going out and doing new things?
4. Been a very nervous person?	5. Do you often get bored? Plus: No diagnosis of depression (Sangha)
5. Felt calm and peaceful?	

NHS = Nurses’ Health Study. DO-HEALTH = Vitamin D3 - Omega3 - Home Exercise - Healthy Aging and Longevity Trial. Sangha = Sangha`s self-administered comorbidity questionnaire. TICS= Telephone Interview for Cognitive Status. MoCA = Montreal Cognitive Assessment. SF-36 = Short Form 36 Health Survey Questionnaire. MHI = Mental Health Index. GDS-5 = Geriatric Depression Scale. * Exclusion criteria of DO-HEALTH. † Not assessed, but severe cases unlikely to be included as seniors with advanced mobility limitations were not included in DO-HEALTH. ± Substitutes, not matching corresponding items from the NHS definition but picturing elements of the NHS requirements regarding disabilities and physical functions. § In analogy to the TICS cutoff, separating seniors with normal cognition from those with mild cognitive impairment (Brand et al. Ciesielska et al.)

Based on the Sangha self-administered comorbidity questionnaire which included diabetes, lung, heart and kidney disease, [40] and considering exclusion criteria from the trial (no cancer, no mobility impairments).

(2) no impairment in cognitive function

We used the Montreal Cognitive Assessment questionnaire (MoCA) [41], with a cut off score of ≥25 as suggested by a recent meta-analysis of 20 studies among community-dwelling seniors [42]

(3) no mental health limitations

We required healthy agers to score < 2 points in the Geriatric Depression Scale short form, comprising five questions (GDS-5), [43] and no self-reported diagnosis of depression, ascertained by the Sangha [44]

(4) no disabilities

We used the Patient Reported Outcome Measurement Information Questionnaire (PROMIS) [45], to evaluate basic and instrumental activities of daily living as well as higher physical function. We required that healthy agers had no limitation in simple activities (e.g. dressing, climbing 5 steps) and no more than moderate limitations in more complicated activities (e.g. running errands and shopping).

### Ascertainment of covariates

Body mass index (BMI) was ascertained dividing participants’ weight in Kg (assessed wearing not more than underwear) by their height squared in meters (assessed without shoes). Physical function was ascertained with the timed repeated sit-to-stand task and with a test of grip strength [46]. In the timed repeated sit-to-stand test, participants had to rise up 5 times from a chair without the help of their arms. Grip strength was measured in Kilo Pascal (KPa) with the Martin Vigorimeter (KLS Martin Group, Tuttlingen Germany). We used mean values from three attempts performed with the dominant hand. Formal education was assessed in total years of formal education completed. All measurements were performed by trained study nurses following standardized procedures in each site.

### Statistical analysis

Baseline characteristics of DO-HEALTH participants are presented as means (standard deviations) for continuous variables and frequencies (percentages) for categorical variables and stratified by country. We conducted ANOVA and chi square tests for comparisons of all characteristics by center.

The prevalence of the HA definition and its components was evaluated as percentages and stratified by age groups (70-74, 75-79 and 80 years and older) and countries. To evaluate that the differences in prevalence between countries were not influenced by age, we also present for each country the age-adjusted prevalence using a logistic regression model with HA as the outcome and age and country as predictors. In addition, we compared the characteristics of participants with HA vs. those without (age, BMI, education, faller status, timed sit-to-stand performance, grip strength, gait speed) using logistic regression adjusting each variable for each other and adjusting for center.

## Results

### Baseline characteristics of participants

The total number of participants with complete data on all domains required to define HA by the NHS tool was 2,123. Among them, 1,306 (61.5%) were women. Participants’ mean age was 74.9 (4.4) years. The mean number of comorbidities assessed by Sangha questionnaire was 1.7 (1.4) and mean MMSE and MoCA scores were 28.5 (1.5) and 25.7 (3.3) respectively. Women had worse physical function with regard to both repeated sit-to-stand and gait speed findings (Table 2).

**Table 2:** Characteristics of participants by country

	Total	Austria	France	Germany	Portugal	Switzerland
	(*n*=2123)	(*n*=199)	(*n*=281)	(*n*=348)	(*n*=297)	(*n*=998)
Age, years	74.9 (4.4)	74.1 (4.1)	75.2 (4.3)	73.3 (2.7)	76 (5)	75.1 (4.6)
Women, N (%)	1306 (61.5%)	102 (51.3)	165 (58.7)	246 (70.7)	189 (63.6)	604 (60.5)
BMI, kg/m^2^						
Men	26.6 (3.5)	25.5 (3.3)	26.8 (3.2)	26.7 (3)	28 (3.5)	26.4 (3.6)
Women	26.2 (4.7)	25 (4.4)	25.1 (4.3)	26.9 (4.7)	29.1 (4.4)	25.5 (4.5)
Mean number of comorbidities	1.7 (1.4)	1.5 (1.3)	2.0 (1.4)	1.7 (1.3)	2.6 (1.7)	1.4 (1.2)
MoCA score	25.7 (3.3)	26.9 (2.5)	27 (2.3)	25.3 (2.3)	21.9 (4.3)	26.3 (2.8)
GDS-5 score	1.8 (2.3)	1.3 (1.4)	2.4 (2.4)	1.2 (1.5)	4.1 (3.6)	1.2 (1.6)
Education, years	12.7 (4.3)	12 (3.7)	13.3 (3.9)	14.5 (3.3)	7.9 (5.4)	13.4 (3.4)
Prior fall, N (%)	884 (41.6)	98 (49.3)	118 (42)	124 (35.6)	120 (40.4)	424 (42.5)
Sit-to-stand, s						
Men	11.2 (4)	8.8 (2.9)	13.5 (3.3)	9.5 (2.1)	15.6 (5.7)	10.4 (2.9)
Women	11.9 (4.3)	10.2 (3.0)	14.4 (4.2)	9.3 (2.3)	17 (5.6)	11.0 (3.0)
Gait speed, m/s						
Men	1.15 (0.22)	1.17 (0.22)	1.14 (0.22)	1.31 (0.18)	1.10 (0.23)	1.12 (0.22)
Women	1.10 (0.23)	1.12 (0.22)	1.06 (0.19)	1.26 (0.2)	0.93 (0.23)	1.09 (0.21)

Values are means and standard deviations unless otherwise noted. *Y* years, *BMI* Body Mass Index, *MoCA* Montreal cognitive Assessment, GDS-5 = Geriatric Depression Scale short form, s = seconds, m/s = meters per second

### Components of the HA definition

For the total study population and based on the four components of the NHS HA definition, 77.6% of participants reported absence of major chronic diseases, 73.3% reported no disabilities in simple tasks and no more than moderate disabilities in complex tasks, 69.9% reported no cognitive impairment and 84.7% had no mental health problems. Healthy agers had a mean MoCA score of 27.5 (SD=1.6) and mean GDS-5 of 0.84 (SD=1.06) whereas the non-healthy agers had a mean MoCA score of 24.4 (SD=3.6) and GDS-5 of 2.44 (SD=2.72).

### Prevalence of HA overall, and by age and country

**Fig. 1:**
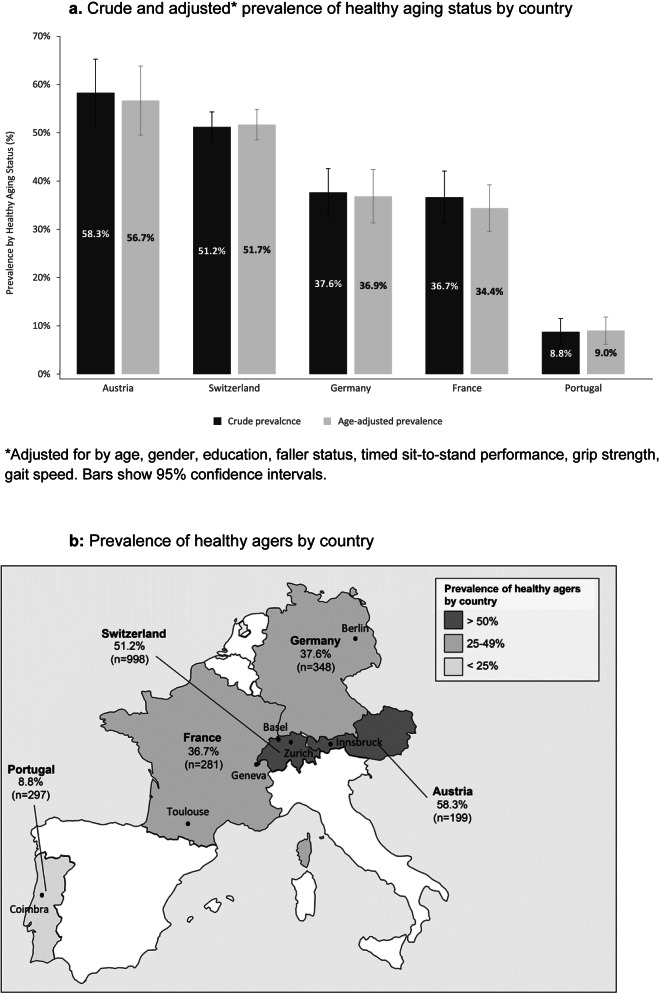
**a** Crude and adjusted* prevalence of healthy aging status by country. *Adjusted for by age, gender, education, faller status, timed sit-to-stand performance, grip strength, gait speed. Bars show 95% confidence intervals. **b** Prevalence of healthy agers by country

In total, 41.8% of participants (887/2123) met all four requirements and thereby qualified as healthy agers. By country, participants from Austria had the highest prevalence of HA with 58.3%, followed by participants from Switzerland 51.2%, Germany 37.6% and France 36.7% whereas the prevalence of HA in participants from Portugal was the lowest with 8.8% (*p* <.0001). (Fig. 1a and b). After adjustment by age, Austria and Switzerland remained the countries with highest prevalence of HA and Portugal was the lowest (Fig. 1a).

**Fig. 2:**
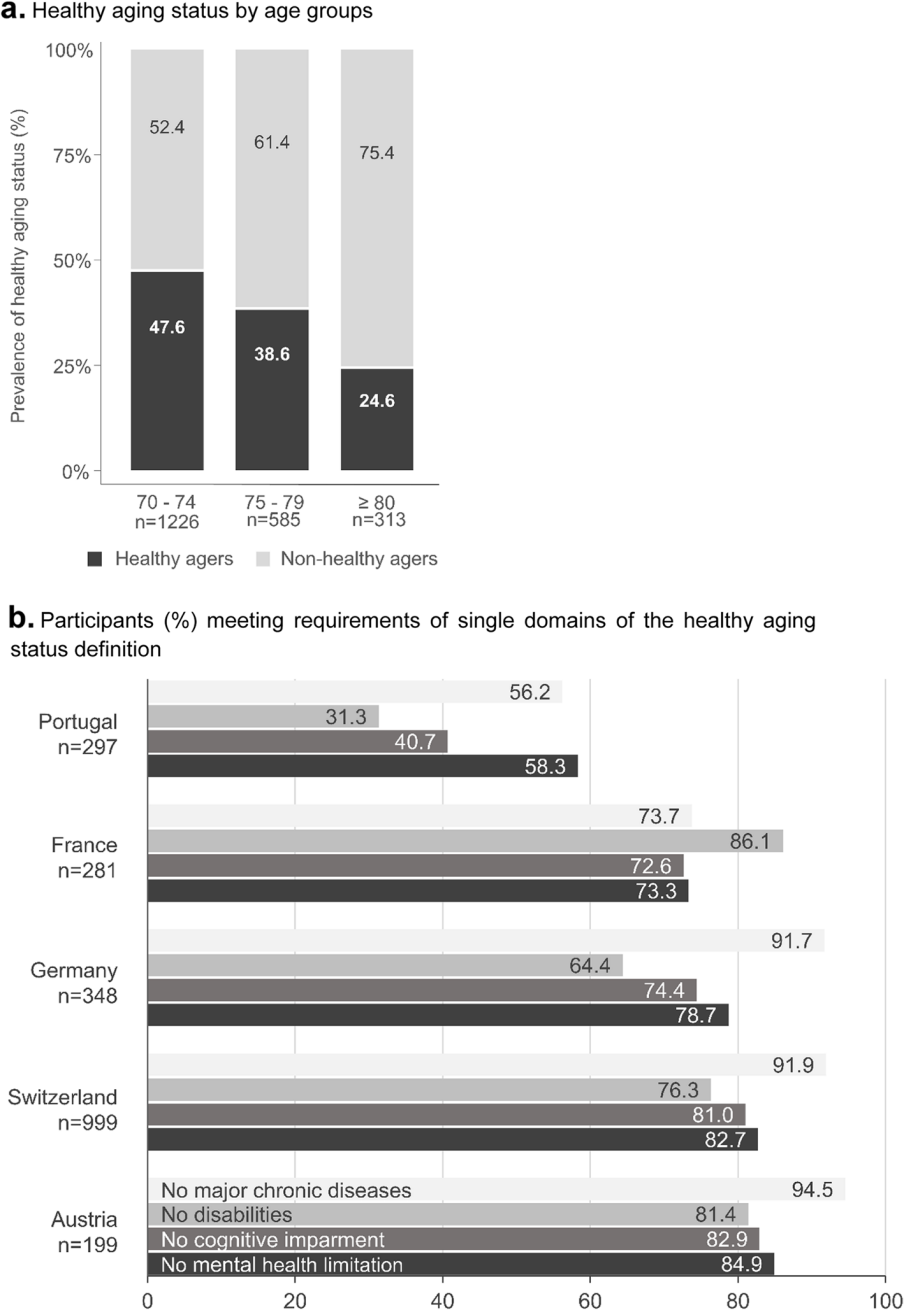
**a** Healthy aging status by age groups. **b** Participants (%) meeting requirements of single domains of the healthy aging status definition

The prevalence was significantly higher in younger participants (47.6% among those 70 to <75 years, 38.7% among 75 to <80 years and 24.6% among those 80 years and older, p<0.0001) (Fig. 2a).

### Performance in the four single components of the HA definition by country

To qualify for HA according to the NHS definition, participants needed to meet the specific requirements of all 4 domains (no major chronic disease, no cognitive impairment, no disabilities, no mental health limitation). However, it is important to show, how many participants in each country met the single requirements of the definition (Fig. 2b). The prevalence of HA can never be higher than the lowest value a population meets in a single domain (bottle neck-phenomenon of multi-dimensional definitions). With regard to the four single components of the NHS HA definition, participants from each country showed a differential performance profile. (Fig. 2b).

### Characteristics of healthy agers

In the unadjusted analyses, healthy agers were on average younger (74.0 vs. 75.5 years, P < .0001), had more years of education, (13.4 vs 12.1, *P* < .0001), had a lower BMI (25.3 vs. 27.1 kg/m^2^, *p* < .0001) and had better physical function (faster gait speed, higher grip strength, shorter time in the sit-to-stand test, and less likely to have a prior fall) (Table 3).

**Table 3:** Characteristics associated with healthy aging status

	Healthy Agers^**a**^ *n*=887	Non-Healthy Agers^**a**^ *n*=1236	***p***-value†	Multivariate model±	
				OR (95%CI)	***p***-value
Age, years	74.0 (3.8)	75.5 (4.7)	< .0001	0.95 (0.93,0.98)	.0001
Women [%]	61.3%	61.6%	0.88	1.36 (1.03,1.81)	0.0319
Education, years	13.4 (3.5)	12.1 (4.7)	< .0001	1.02 (0.99,1.05)	0.233
Prior fall [%]	38.9%	42.6%	0.03	0.87 (0.72,1.07)	0.1813
BMI, Kg/m^2^	25.3 (3.8)	27.1 (4.4)	< .0001	0.94 (0.91,0.96)	< .0001
Gait speed, m/s					
Men	1.20 (0.20)	1.12 (0.23)	< .0001	4.70 (2.68,8.25)	< .0001
Women	1.18 (0.20)	1.04 (0.24)	< .0001		
Grip strength, KPa					
Men	79.0 (15.7)	73.4 (17.5)	< .0001	1.01 (1.00,1.01)	0.2655
Women	53.2 (10.5)	48.9 (12.3)	< .0001		
Sit-to-stand, s					
Men	10.0 (2.9)	12.1 (4.5)	< .0001	0.90 (0.87,0.93)	< .0001
Women	10.3 (2.7)	13.1 (4.9)	< .0001		

^a^Values are means and SE unless noted. † *p*-values are from T-tests or X^2^ tests

±Variables in the multivariate model are adjusted for each other and for center

In the multivariate adjusted model, younger age, female gender, lower BMI, faster gait speed and a shorter time in sit-to-stand test were independently associated with a prevalent HA. For every additional year of age, participants had 5% lower odds of being healthy agers (OR = 0.95, 95% CI 0.93 to 0.98, P = .0001) and for every additional BMI point, participants had 6% lower odds of being healthy agers (OR = 0.94, 95% CI 0.91 to 0.96, *P* = < .0001). Women had 36% higher odds of being healthy agers (OR = 1.36, 95% CI 1.03 to 1.81, *P* = .0319). For every meter per second increase in gait speed participants had 4-times higher odds of being healthy agers (OR = 4.70, 95% CI 2.68 to 8.25, *P* = < .0001) but for every second increase in the sit-to-stand test, participants had 10% lower odds of being healthy agers (OR = 0.90, 95% CI 0.87 to 0.93, *P* = < .0001). Years of education, prior falls and grip strength where not independently associated with HA (Table 3).

## Discussion

In this large cross-sectional study, we examined 2,123 DO-HEALTH trial participants recruited from 5 countries pre-selected to be relatively healthy older adults age 70 years and older. On average, 41.8% of participants met the HA criteria, but there were significant differences between countries. Prevalence of HA was highest in participants from Switzerland (51.2%) and Austria (58.3%) and lowest in participants from Portugal (8.8%). At the cross-sectional level, HA was independently and significantly associated with younger age, female gender, lower BMI and better physical function regarding gait speed and sit-to-stand test. Notably, however, education, prior falls and grip strength were not independently associated with HA.

Similar to our findings, results from a population-based study, the Survey of Health, Aging and Retirement in Europe (SHARE) revealed substantial variability in HA prevalence between countries ranging from 21.1% in Denmark to 1.6% in Poland among adults with a mean age of 72 (6.7) years [9]. These differences support variations in health states of European older adults by country also at the population-based level. Given the pre-selection of relatively healthy adults with good mobility and cognitive function and no major health events in the 5 years prior to recruitment, DO-HEALTH compared with SHARE, shows a higher prevalence of HA in community-dwelling European adults age 70 and older. In addition, HA in SHARE was operationalized according to the definition established by Mc Laughlin [8]. This HA definition shares numerous similarities with the NHS definition however, considerable differences exist. Especially the incorporation of the HA domain of “social engagement” is of importance with regard to the lower prevalence of HA in SHARE. Being met by only 27.1% of participants, it created a bottleneck, a well-known challenge of HA definitions with higher numbers of domains or stricter cut-off values.

In our study, based on the NHS definition, DO-HEALTH participants from Portugal had the lowest prevalence of HA in comparison to all other countries, despite the same inclusion and exclusion criteria and even after age adjustment. Consistent with our results, SHARE reported a lower prevalence of HA in southern countries of Europe (Italy 5.3%; Greece 7.7%; Spain 3.1%) compared to countries like Austria 10.2%, Switzerland 16.1% or Germany 11.6% [9]. The observed difference in the prevalence of HA between participants from Portugal and the five other European countries in DO-HEALTH could potentially be explained by differences in socioeconomic status [47]. The available median equivalent purchasing power per capita in Portugal in 2016 was around half as much as in France, Germany and Austria, and only round one third as much as in Switzerland [47]. Consistently, in DO-HEALTH, years of education as a surrogate to income, differed between countries and were lowest in participants from Portugal (mean = 8.0, SD = 5.4 years) and highest in participants from Germany (mean = 14.5, SD = 3.3 years). However, education was not independently associated with the total score of the HA definition in DO-HEALTH.

With regard to age, DO-HEALTH reflects a decline in HA with age even among this relatively healthy selection of older adults from 47.6% to 38.7% to 24.6 % in adults 70-74, 75-79 and more than 80 years of age, respectively. While this is best explained by a higher incidence of chronic diseases, disabilities, cognitive impairment and mental health limitations with advanced age, our data also supports the potential of being a healthy ager even at age 80 and older in one out of four cases.

With regard to gender, we found an independent association between HA and female gender. Reports from the Organisation for Economic Co-operation and Development (OECD) as well as results from European studies of gender differences have repeatedly shown advantages concerning life expectancy, ischemic heart diseases, cancer and general health status for women compared with men [48–50].

In support of the important role of physical activity in overall health and aging, HA in DO-HEALTH was independently associated with faster gait speed and better performance in the sit-to-stand. These results are in accordance with the prognostic benefits of maintained physical function in older age described in the literature. Pooled analysis of individual data from nine older adult cohorts (34,485 community-dwelling seniors) showed an independent association between faster gait speed and increased survival across all gait speeds and age groups [51]. In addition, prospective data suggest, that an increase in gait speed on an individual level predicts increased survival, strengthening the preventative role of physical function [52]. Also, better performance in the sit-to-stand test has been independently associated with better physical function and increased survival [53, 54].

Further, DO-HEALTH suggests that for every additional BMI point, older adults may have 6% lower odds of being healthy agers. In fact, healthy agers showed, on average, BMI levels around the upper limit of normal (25 [3.8] Kg/m^2^), and non-healthy agers were mildly overweight (27.1 [4.4] Kg/m^2^). These findings are consistent with the literature, where a normal BMI (18.5 - 24.9 Kg/m^2^) is associated with reduced mortality compared to the BMI values in the overweight and obese spectrum [55–58]. Alternatively, in unselected older adults, BMI level above normal have been associated with reduced mortality [59–62]. The optimal BMI for older adults is not known, optimal BMI level between 20 and 29.9 Kg/m^2^ have been described [61]. DO-HEALTH might suggest that among relatively healthy older adults, the upper normal range may be most advantageous for HA, however, prospective investigations are needed to determine an optimal BMI in this population.

Our study has several strengths. We used a well-validated NHS definition of HA, extracted from standardized clinical health assessments derived from the baseline examination of a large clinical trial. Further, our study reflects extremely well phenotyped adults age 70 and older from 5 European countries including both southern and central Europe. The observed differences in HA are conservative as we targeted relatively healthy older adults, and the observed pattern of a lower HA prevalence in southern Europe and specifically Portugal is supported by the literature [9, 63]. However, it is noteworthy that the cities and samples included in each study do not represent the entire country.

Our study also has limitations. First, DO-HEALTH is not a population-based study. Results of the trial participants are not representative of the prevalence in HA and the functional abilities of the general population in the 5 respective countries of the DO-HEALTH centres. Our study population reflect a sample of relatively healthy older adults in a rigorous clinical trial setting. Second, even with the same inclusion and exclusion criteria applied in all 5 countries, bias due to different priorities in defined recruitment strategies between centers cannot be completely excluded. Last, the cross-sectional nature of our analysis does not allow us to draw a causal relationship between the covariates explored for their association with HA.

## Conclusions

In conclusion, in this sample of pre-selected relatively healthy European adults age 70 and older participating in the DO-HEALTH trial, the prevalence of HA differed across five countries with the lowest prevalence in Portugal compared with Austria, Switzerland, Germany and France. Independent of country, prevalence of HA was associated with younger age, lower BMI, female gender and better physical function. Further studies are needed to examine differences in HA between European countries at the population-based and prospective level.

## Supplementary Information

**Additional file 1.** Detailed description of the operationalization of the Nurses` Health Study healthy aging definition in DO-HEALTH.

## Data Availability

The datasets used and/or analysed during the current study are available from the corresponding author on reasonable request.

## Abbreviations

HA: Healthy Aging
NHS: Nurses` Health Study
MoCA: Montreal Cognitive Assessment
MMSE: Mini Mental Status Examination
PROMIS: Patient Reported Outcome Measurement Information Questionnaire
SHARE: Survey of Health, Aging and Retirement in Europe
