# Supplementary material for: Prevalence of healthy aging among community dwelling adults age 70 and older from five European countries
Source: BMC Geriatr. 2022 Mar 2;22:174. doi: 10.1186/s12877-022-02755-8 (PMC8889763; doi:10.1186/s12877-022-02755-8)
Supplement: Supplementary file 1 — Additional file 1. Detailed description of the operationalization of the Nurses` Health Study healthy aging definition in DO-HEALTH. [file 12877_2022_2755_MOESM1_ESM.docx]

**Supplementary material**

**Detailed description of the operationalization of the Nurses` Health Study healthy aging definition in DO-HEALTH**

No Major Chronic Diseases

The NHS definition of healthy aging (HA) classified healthy agers to be free of 11 major chronic diseases: (1) type 2 diabetes, (2) congestive heart failure, (3) kidney failure, (4) chronic obstructive pulmonary disease, (5) cancer other than non-melanoma skin cancer, (6) myocardial infarction, (7) coronary artery bypass surgery, or angioplasty, (8) Stroke (9) Parkinson’s disease, (10) multiple sclerosis, and (11) amyotrophic lateral sclerosis.

In DO-HEALTH, we used the Sangha`s self-administered comorbidity questionnaire, ^1^ to identify participants free of (1) type 2 diabetes, (2) congestive heart failure, (3) kidney failure and (4) chronic obstructive pulmonary disease, . In addition, DO-HEALTH participants were free of (5) cancer other than non-melanoma skin cancer, (6) myocardial infarction, (7) coronary artery bypass surgery, or angioplasty and (8) Stroke, as these were exclusion criteria of the trial. In contrast to the NHS, we did not directly asses (9) Parkinson’s disease, (10) multiple sclerosis, and (11) amyotrophic lateral sclerosis. However, seniors with hemiplegia, severe gait impairment, those who could not walk 10 meters or could not get in and out of a chair without help, were not included in DO-HEALTH. Therefore, it is unlikely that participants of DO-HEALTH suffered from advanced stages of these neurological conditions.

No Disabilities

The NHS used questions from the Short Form 36 Health Survey Questionnaire (SF-36),^2^ to classify healthy agers with regard to disabilities. Participants were defined as healthy agers if they had no limitation on the moderate activities of (1) bathing, (2) dressing, (3) climbing one flight of stairs, (4) walking >1 mile or walking several blocks, (5) moving a table, (6) bowling or playing Golf, or (7) pushing a vacuum cleaner. In addition, it was required to have no more than moderate limitations on more demanding physical performance measures like (8) lifting or carrying groceries, (9) bending, kneeling, or stooping, (10) lifting heavy objects and (11) climbing several flights of stairs.

In DO-HEALTH, we matched all of the above listed items (1) - (11) using the Patient Reported Outcome Measurement Information Questionnaire (PROMIS).^3^ The PROMIS is a self-administered tool comprising basic and instrumental activities of daily living as well as higher physical function. The questionnaire includes 20 items with a range between 0 (without any difficulties) and 4 (unable to do). Based on the PROMIS, we were able to match items 1-4 requiring no difficulties in (1) “Wash and dry your body” (substitute for “bathing”), (2) “Dress yourself, including shoelaces and buttons” (substitute for “dressing”), (3) “Climb up five steps” (substitute for “climbing one flight of stairs”) and (4) “Walk a block on flat ground” (substitute for “walking >1 mile or walking several blocks”). For items (5) - (7), that could not be directly matched with identical items, we chose “Standing up from an armless straight chair”, “Getting in and out of bed”, and “Getting on and off the toilet” to select participants with comparable physical functions within this group of questions demanding no limitation in basic and moderate activities. To match the more demanding physical performance measures from the NHS definition (8 - 11), we allows “no more than some difficulties” in (8) “Run errands and shop” (substitute for “Lifting or carrying groceries”), (9) “Bending, kneeling, or stooping” (substitute for “Do chores such as vacuuming or yard work”), (10) “Reach and get down a 2-kilogram object from above your head” (substitute for “Lifting heavy objects”) and (11) “Bend down to pick up clothing from the floor” (substitute for “Climbing several flights of stairs”). The last item (11) was not a perfect match to the NHS item “climbing several flights of stairs”. However, we included this question as, especially in older age, bending down and picking up something from the floor, reflects a more demanding physical performance measure as it was required in the NHS.

No Impairment in Cognitive Function

For the NHS definition, healthy agers needed to score ≥31 out of 41 points in the original Telephone Interview for Cognitive Status (TICS), modeled after the Mini Mental State Examination (MMSE) ^4^.

In DO-HEALTH, we used the the Montreal Cognitive Assessment (MoCA) ^5^ with a cutoff score of ≥25. The MoCA is a validated screening instrument for cognitive decline with high sensitivity for the detection of mild cognitive impairment (MCI) ^6^. The TICS cutoff of ≥31 used in the NHS was originally aimed to separate seniors with normal cognition from those with mild to moderate stages of cognitive impairment ^4^. As data on reliable conversion rates between TICS and MoCA are missing, we used a MoCA cutoff score of ≥25 as suggested by a recent meta-analysis of 20 studies among community-dwelling seniors, where best accuracy for separating those with normal cognition from those with MCI could be achieved with a cutoff point of ≥25 reaching a sensitivity / specificity of 80.5 / 81.2 %.^6^

No Mental Health Limitation

The NHS definition of HA required to score >84 out of 100 points (the median value in the analytic cohort) in the SF-36 Mental Health Index (SF-36 MHI).^7^ Participants had to select, how much of the time during the last month they had (1) “felt so down in the dumps that nothing could cheer them up?”, (2) “been a happy person?”, (3) “felt downhearted and blue?”, (4) “been a very nervous person?”, (5) “felt calm and peaceful?” Answers choices ranged from “all of the time” (1 point) to “none of the time” (6 points); a score of 0-100 points was created.

In DO-HEALTH, we required healthy agers to score < 2 points in the Geriatric Depression Scale short form, comprising five questions (GDS-5). The GDS is a validated questionnaire for mental health among seniors,^8^ and three questions directly relate to the MHI: (1), GDS: “Do you feel pretty worthless the way you are now?” (Substitute for MHI: “felt so down in the dumps that nothing could cheer them up”), (2), GDS: “Are you basically satisfied with your life?” (Substitute for MHI: “been a happy person”) and (3), GDS: “Do you often feel helpless?” (Substitute for MHI: “felt downhearted and blue”). The remaining questions from the MHI (“have you been a very nervous person?” and “have you felt calm and peaceful?) refer to anxiety, which is not well covered by the GDS-5. However, the GDS question (5) “Do you prefer to stay at home rather than going out and doing new things?” partly pictures anxiety-related (social) withdrawal and can therefore be regarded as a substitute for the MHI anxiety questions. In addition to a score of < 2 points in the GDS-5, DO-HEALTH participants, who had a diagnosis of depression, ascertained by the Sangha questionnaire, did not meet the requirements of HA.

**References**

1. Sangha, O, Stucki, G, Liang, MH, et al. The Self-Administered Comorbidity Questionnaire: a new method to assess comorbidity for clinical and health services research. Arthritis Rheum 2003;49(2):156-163.

2. Ware, J. SF-36 Health Survey update*.* 2001.

3. Maska, L, Anderson, J, Michaud, K. Measures of functional status and quality of life in rheumatoid arthritis: Health Assessment Questionnaire Disability Index (HAQ), Modified Health Assessment Questionnaire (MHAQ), Multidimensional Health Assessment Questionnaire (MDHAQ), Health Assessment Questionnaire II (HAQ-II), Improved Health Assessment Questionnaire (Improved HAQ), and Rheumatoid Arthritis Quality of Life (RAQoL). Arthritis care & research 2011;63 Suppl 11:S4-13.

4. Jason Brandt, MS, Marshal Folstein. The Telephone Interview for Cognitive Status. Neuropsychiatry, Neuropsychology and Behavioral Neurology 1988;Vol. 1 Nr. 2:pp. 111-117.

5. Nasreddine, ZS, Phillips, NA, Bedirian, V, et al. The Montreal Cognitive Assessment, MoCA: a brief screening tool for mild cognitive impairment. J Am Geriatr Soc 2005;53(4):695-699.

6. Ciesielska, N, Sokolowski, R, Mazur, E, et al. Is the Montreal Cognitive Assessment (MoCA) test better suited than the Mini-Mental State Examination (MMSE) in mild cognitive impairment (MCI) detection among people aged over 60? Meta-analysis. Psychiatria polska 2016;50(5):1039-1052.

7. Rumpf, HJ, Meyer, C, Hapke, U, et al. Screening for mental health: validity of the MHI-5 using DSM-IV Axis I psychiatric disorders as gold standard. Psychiatry research 2001;105(3):243-253.

8. Hoyl, MT, Alessi, CA, Harker, JO, et al. Development and testing of a five-item version of the Geriatric Depression Scale. Journal of the American Geriatrics Society 1999;47(7):873-878.
